# Supplementary material for: Acute and Long-Term Outcomes of ST-Elevation Myocardial Infarction in Cancer Patients, a ‘Real World’ Analysis with 175,000 Patients
Source: Cancers (Basel). 2021 Dec 9;13(24):6203. doi: 10.3390/cancers13246203 (PMC8699199; doi:10.3390/cancers13246203)

Supplemental Material

File S1

Material and Methods (comprehensive information)

The German remuneration system is based on the system of the "German Diagnosis Related Groups" (G-DRG). This requires the coding of a main diagnosis for all inpatients, which must be carefully selected after discharge, taking into account the underlying reason for hospital admission. In addition, an unlimited number of secondary diagnoses can be coded to reflect co-morbidities and complications, whether present or during the hospital stay. These secondary diagnoses increase the patient's comorbidity and complexity and have some influence on reimbursement. Each diagnosis has to be coded according to the "German Modification of the International Statistical Classification of Diseases and Related Health Problems 10th Revision" (ICD-10 GM). In addition to the WHO ICD-10, some diagnoses are more detailed in the German Version due to the coding requirements of the G-DRG-System. This enables the separation of subgroups such as various types of cancer, as well as acute ST-segment elevation myocardial infarction (STEMI) and non-STEMI. Similar to the ICD for diagnosis, all diagnostic, endovascular and surgical procedures must be coded according to the German procedure classification ("Operationen und Prozedurenschlüssel", OPS). Most of them have direct impact on reimbursement.

Using the coded diagnoses and procedures, each case is then assigned to a specific G-DRG, depending on its main diagnosis and combination of secondary diagnoses and procedures, and induces a certain reimbursement of costs.

All applied ICD-10 GM and OPS codes are listed in **Supplemental Table S1.**

Due to the high influence of diagnoses and procedures on reimbursement, around 30% of all cases are checked and corrected by independent medical working groups ("Medizinischer Dienst").

Data Source

The Allgemeine Ortskrankenkasse (AOK) is an amalgamation of 16 regional health insurances, which together form the largest statutory health insurance in Germany. The AOK is currently responsible for more than 26 million people, around 30% of the entire German population. All patient data is stored in a central IT database of the AOK Research Institute (WIdO, Berlin), from which we receive aggregated and anonymized data of all patients who meet the following criteria.

Patient selection

All patients with an age ≥18 years were hospitalized with a coded main diagnosis STEMI (ICD-10 GM code I21.0, I21.1; I21.2, I22.0, I22.1, I22.8) in 2010 up to 2017 were included in the analysis (**Supplemental Figure S1**). This hospitalization was defined as the index hospitalization. Patients were divided into different cancer subgroups according to a cancer diagnosis during the Index hospital stay or in the previous 2 years, as follows: colon cancer (C18*-C21*), lung cancer (C34*), skin cancer (C43*-C44*), breast cancer (C50*), prostate cancer (C61*), urinary tract cancer (C64-C68*), any other cancer (C00*-C97*, except the forementioned cancer types), and no cancer (no C* code in history); **Supplemental Figure S2**. The baseline characteristics also included other coded diagnoses, such as hypertension, diabetes mellitus, dyslipidemia, atrial fibrillation, metastasis at or within two years of index hospitalization, and procedures such as percutaneous coronary intervention within two years of index hospitalization.

In-hospital treatment, outcome, medication and follow-up

All coded interventions during the hospital stay as well as the diagnoses shock, death, stroke, bleeding, sepsis and acute kidney failure were considered as inpatient treatment or outcome. Patients were followed up for up to nine years after index hospitalization.

Statistical methods

Patient data were grouped by the cancer type that was coded for descriptive analysis during their baseline phase. In order to obtain different patient groups, the grouping was carried out in a hierarchical manner, which is shown in the **Supplementary Figure S2**. Patients with breast cancer were placed in the breast cancer group if they had breast cancer and no prostate cancer. Patients were placed in the lung cancer group if they had lung cancer and no prostate or breast cancer, etc. The exact hierarchical order was prostate cancer> breast cancer> lung cancer> colon cancer> urinary tract cancer> skin cancer> no cancer. Qualitative data were tested with a two-tailed chi-square test and quantitative data with a two-tailed Wilcoxon test. The 8-year overall survival rate (OS) was estimated using a Kaplan-Meier estimator; all p-values of the test procedures described above are purely descriptive and not adjusted.

The endpoint OS was analyzed using the multivariable Cox regression model. The models included baseline patient risk profiles. In contrast to the descriptive analysis, the patients were not divided into different cancer groups and each patient can have several types of cancer in his risk profile. All presented 95% confidence intervals (CIs) and p-values are by default unadjusted and purely descriptive. The hazard ratios (HRs) and the unadjusted 95%CI for all characteristics are shown in the tables and figures. All analyzes should be fully exploratory

(generating hypotheses), non-confirmatory and will be interpreted accordingly. Statistical analyzes were carried out with R version 3.6.0 (2019-04-26), R foundation, Vienna, Austria.

Data Accessibility

The authors confirm that the data utilized in this study cannot be made available in the manuscript, the supplemental files, or in a public repository due to German data protection laws ('Bundesdatenschutzgesetz', BDSG). Therefore, they are stored on a secure drive in the AOK Research Institute (WIdO), to facilitate replication of the results. Generally, access to data of statutory health insurance funds for research purposes is possible only under the conditions defined in German Social Law (SGB V § 287). Requests for data access can be sent as a formal proposal specifying the recipient and purpose of the data transfer to the appropriate data protection agency. Access to the data used in this study can only be provided to external parties under the conditions of the cooperation contract of this research project and after written approval by the sickness fund. For assistance in obtaining access to the data, please contact wido@wido.bv.aok.de.

Ethical vote

The data available here were evaluated in the GenderVasc research project. This project was approved by the ethics committee of the Landesaerztekammer Westfalen-Lippe and the medical faculty of the Westphalian Wilhelms University of Muenster (No 2019-21-f-S).

Supplemental Table S1: Codes for data retrieval

**International classification of disease 10th Revision, German Modification (ICD-10 GM)**	**Code**
Acute STEMI	I21.0, I21.1; I21.2, I22.0, I22.1, I22.8
Previous myocardial infarction (MI)	I21.-; I22.-
Cerebrovascular Disease (CVD)	I65.-; I66.-; I67.2
Previous stroke	I63.-; I64.-; I69.3; I69.4
Hypertension	I10.-; I11.-; I12.-; I13.-; 15.-
Diabetes mellitus (DM)	E10.-; E11.-; E12.-; E13.-; E14.-
Dyslipidemia	E78.-
Obesity	E66.-
Smoking	F17.-
Artrial fibrillation (AF) and flutter (AFL)	I48.-
PAD 1-3	I70.20; I70.21; I70.22 (from 2015)
PAD 4-6	I70.22 (until 2015);<br>I70.23; I70.24; I70.25 (from 2015)
Chronic heart failure (CHF)	I50.-
Chronic kidney disease (CKD)	N18.- ; N19.-
Cancer	C00.- to C97.-
Colon Cancer	C18.-; C19.-; C20.-; C21.-
Lung Cancer	C34.-
Skin Cancer	C43.-; C44.-
Breast Cancer	C50.-
Prostate Cancer	C61.-
Urinary Tract Cancer	C64.-; C65.-; C66.-; C67.-; C68.-
Diseased Coronary Vessels: 1	I25.11 (and no I25.12 or I25.13)
Diseased Coronary Vessels: 2	I25.12 (and no I25.13)
Diseased Coronary Vessels: 3	I25.13
Shock	T81.1; R57.0
Acute stroke	I63.-; I64.-
Hemorhagic stroke	I60.-; I61.-; I62.-
Bleeding	K92.-; H44.8; T81.0; T81.2; T81.3; T81.7
Sepsis	A41;
Acute Renal Failure (ARF)	N17;
Previous CABG	Z95.1 (with additional OPS codes)
Previous heart Valve implantation	Z95.2 (with additional OPS codes)
Metastasis	C77.-; C78.-; C79.-
**German procedure classification system (OPS)**	
Blood transfusion	8-800.0; 8-800.1; 8-800.c
PCI (previous or current)	8-837;
Current CABG	5-36 (with additional ICD codes)
Current valve implantation	5-351; 5-352; 5-353; 5-354 (with additional ICD codes)

Abbreviations:

AF, atrial fibrillation; AFL, atrial flutter; ARF, Acute renal failure; CABG, coronary artery bypass grafting; CHF, chronic heart failure; CKD, chronic kidney disease; CVD, cerebrovascular disease; DM, diabetes mellitus; MI, myocardial infarction; OPS, Operationen und Prozedurenschluessel; PAD, peripheral artery disease; PCI, percutaneous coronary intervention; STEMI, ST-elevation myocardial infarction.

Supplemental Figure S1:

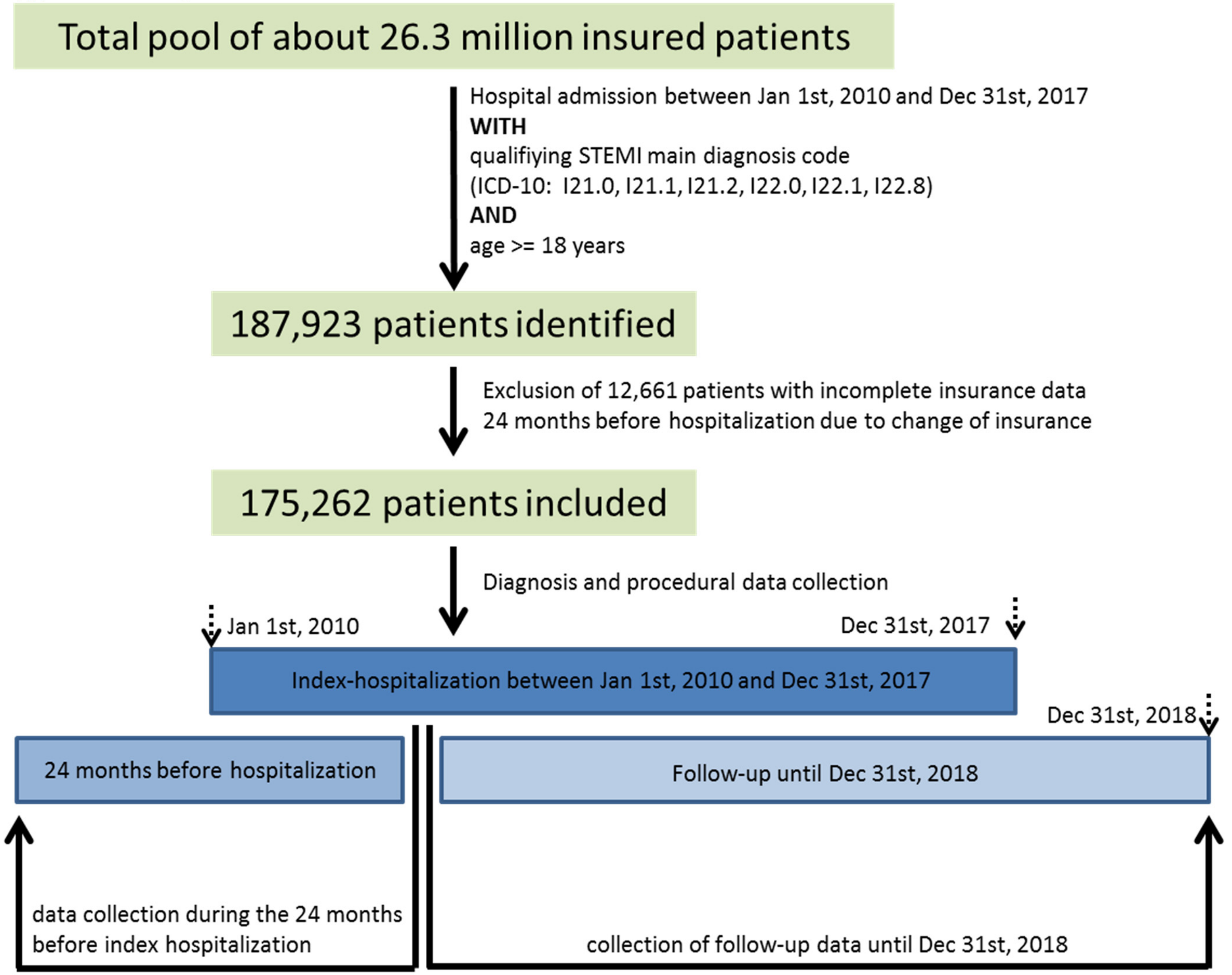

Supplemental Figure S2:

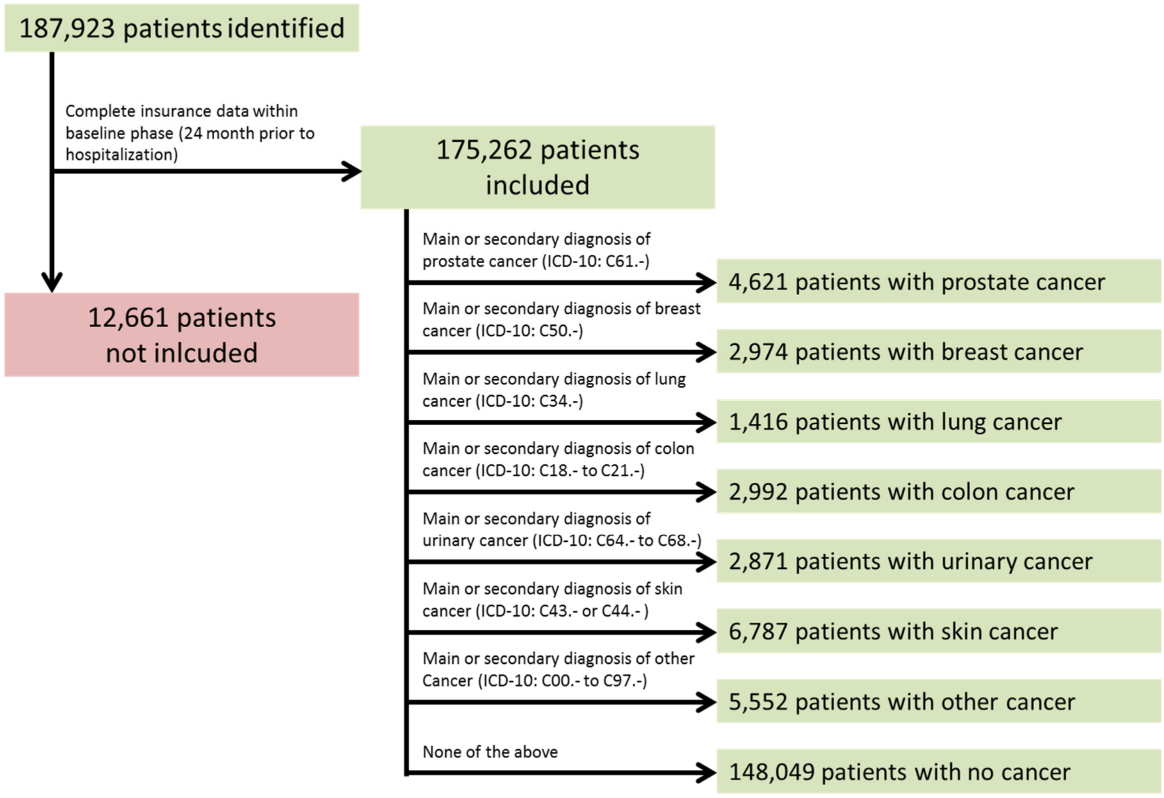

Supplement: Supplementary file 1 [file cancers-13-06203-s001.zip › cancers-1483650-supplementary.pdf]
